# Tocotrienols induce endoplasmic reticulum stress and apoptosis in cervical cancer cells

**DOI:** 10.1186/s12263-016-0543-1

**Published:** 2016-12-23

**Authors:** Raffaella Comitato, Barbara Guantario, Guido Leoni, Kalanithi Nesaretnam, Maria Beatrice Ronci, Raffaella Canali, Fabio Virgili

**Affiliations:** 1 Council for Agricultural Research and Economics - Food and Nutrition Research Centre (C.R.E.A.-AN), via Ardeatina 546, 00178 Rome, Italy; 2 Department of Physics, Sapienza University of Rome, Piazzale Aldo Moro 5, 00185 Rome, Italy; 3 Malaysian Palm Oil Board, 6 Persiaran Institusi, Bandar Baru Bangi, 4300 Selangor, Malaysia

**Keywords:** Tocotrienol, Endoplasmic reticulum stress, XBP-1, Apoptosis, IRE-1α

## Abstract

**Background:**

We have previously reported that γ- and δ-tocotrienols (γ- and δ-T3) induce gene expression and apoptosis in human breast cancer cells (MDA-MB-231 and MCF-7). This effect is mediated, at least in part, by a specific binding and activation of the estrogen receptor-β (ERβ). Transcriptomic data obtained within our previous studies, interrogated by different bioinformatic tools, suggested the existence of an alternative pathway, activated by specific T3 forms and leading to apoptosis, also in tumor cells not expressing ER. In order to confirm this hypothesis, we conducted a study in HeLa cells, a line of human cervical cancer cells void of any canonical ER form.

**Results:**

Cells were synchronized by starvation and treated either with a T3-rich fraction from palm oil (10–20 μg/ml) or with purified α-, γ-, and δ-T3 (5–20 μg/ml). α-tocopherol (TOC) was utilized as a negative control. Apoptosis, accompanied by a significant expression of caspase 8, caspase 10, and caspase 12 was observed at 12 h from treatments. The interrogation of data obtained from transcriptomic platforms (NuGO Affymetrix Human Genechip NuGO_Hs1a520180), further confirmed by RT-PCR, suggested that the administration of γ- and δ-T3 associates with Ca^2+^ release. Data interrogation were confirmed in living cells; in fact, Ca-dependent signals were observed followed by the expression and activation of IRE-1α and of other molecules involved in the unfolded protein response, the core pathway coping with endoplasmic reticulum stress in eukaryotic cells, finally leading to apoptosis.

**Conclusions:**

Our study demonstrates that γ- and δ-T3 induce apoptosis also in tumor cells lacking of ERβ by triggering signals originating from endoplasmic reticulum stress. Our observations suggest that tocotrienols could have a significant role in tumor cell physiology and a possible therapeutic potential.

**Electronic supplementary material:**

The online version of this article (doi:10.1186/s12263-016-0543-1) contains supplementary material, which is available to authorized users.

## Background

The chemical structure of tocotrienols (T3) is very similar to that of tocopherols (TOC), only differing in the unsaturation of the phytyl chain. On the basis of a modest inhibitory effect in a fetal resorption test in rats, T3 are frequently pooled together with TOC within the family of “vitamin E,” but a wide spectrum of specific biological activities has also been reported that is not exhibited by TOC [27]. For instance, several studies have demonstrated that T3, especially the γ- and δ-T3 isoforms, have inflammatory and antioxidant activities not shared with TOC and in particular with α-TOC [26, 54, 57]. Moreover, evidences exist indicating that each vitamin E isomer has a specific pharmaco-dynamic profile [3, 6].

In a study based on a transcriptomic (complementary DNA (cDNA) array) approach, we have previously reported that a T3-rich fraction (TRF) extracted from palm oil induces a significant inhibition of cell proliferation both in vitro in cultured breast cancer cells [40, 41] and in vivo in tumors caused by the inoculation of human breast cancer cells in athymic mice [40]. More recently, on the basis of a subsequent set of studies in silico, followed by in vitro binding experiments coupled with cell culture studies, we have demonstrated that the effects of specific T3 (γ- and δ-T3 forms) on gene expression are, at least in part, mediated by the binding to estrogen receptor-β (ERβ) in cultured MDA-MB-231 [16] and MCF-7 cells [15]. The transcriptomic data set obtained within these studies, further interrogated by means of bioinformatic tools, suggested the existence of an alternative pathway, activated by specific T3 forms, leading to apoptosis also in tumor cells not expressing any of the two canonical forms of ER (ERα and ERβ). Data interrogation suggested the hypothesis that this alternative pathway could be mainly ascribable to the induction of a cellular stress, at the level of the endoplasmic reticulum (EndoR).

We have therefore extended our previous investigation exploring a putative pathway activated at the level of EndoR by specific T3 forms. To this aim, HeLa cells, a cell line not expressing any of the canonical forms of ER, were selected as the experimental model. This paper reports the activation of EndoR stress and Ca-dependent gene expression following the administration of T3, leading to apoptosis, independently of the presence of estrogen receptors.

## Results

### TRF and T3 induce apoptosis in HeLa cells

As mentioned in the “Background” section, the interrogation of transcriptomic data collected in our previous investigations [15, 16], further corroborated by recent indication that appeared in the literature [61], suggested the presence of a pro-apoptotic effect of T3, independent on ERβ signaling. Therefore, HeLa cells, void of these receptors, were utilized to verify the hypothesis of a pro-apoptotic effect of T3 in the absence of ER.

**Fig. 1:**
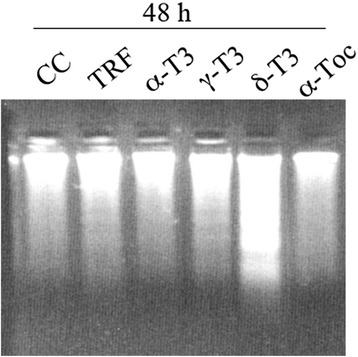
T3-induced apoptosis in HeLa cells. TRF, γ-, and δ-T3 (10 μg/ml) induce apoptosis in HeLa cells as indicated by an evident DNA laddering at 48 h from treatment

First of all, we assessed if T3 and TRF could induce cell death also in tumor cells not expressing ERs. Figure 1 shows that at 48 h from TRF and γ- and δ-T3 administration, cells display DNA laddering followed by cellular death by apoptosis (morphology not shown) in HeLa cells. Noteworthy, the apoptotic effect of T3 in HeLa cells was detectable 24 h later with respect to what we observed in our previous experiments in MCF-7 cells [15], this time shift suggesting the presence of a distinct pathway modulated by T3. The presence of α-TOC and α-T3 were not associated with any detectable DNA laddering.

### Identification and characterization of alternative pathways affected by T3 in HeLa cells

On the basis of this initial observation, we characterized the changes of transcriptome expression of HeLa cells associated with 24 h of T3 treatment by a microarray approach.

The profiles of differentially expressed genes obtained by microarrays were further subjected to functional analysis with the aim of characterizing biological processes and cellular components annotated in GO and modulated by T3.

Finally, by clustering “semantic” similarities [4, 30] between enriched GO terms, we compared the transcriptomic phenotype observed in HeLa with that previously observed in our laboratories on MCF-7 cells treated with T3 [15].

**Fig. 2:**
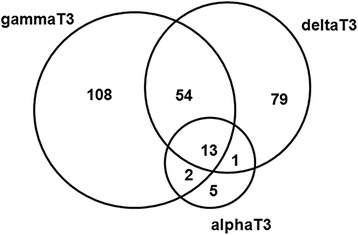
Venn diagram reporting the number of differentially expressed genes observed after treatment of HeLa cells with T3. Differentially expressed genes were identified by LIMMA analysis according to a fold-change threshold (treatment vs control) of at least ±0.5 and a *p* value threshold above 0.05

Microarray analysis indicated that the treatment with 10 μg/ml γ-T3 and δ-T3 is associated with a higher number of modulated genes (177 genes and 147 genes, respectively) than that observed following α-T3 treatment (21 genes) (see Fig. 2 and Additional file 1: Table S1).

Out of 13 genes significantly modulated by all T3, 8 genes have been described to be specifically involved in sterol and steroid metabolism (DHCR24, FADS2, FDFT1, IDI1, INSIG1, LDLR, SCD, SREBF1) and 5 genes (KLF7, LPIN1, FADS2, MMAB and MYO6) have been reported to play a role in different aspects of cellular metabolism. More specifically, KLF7 is involved into regulation of adiponectin expression in mouse embryonic fibroblast [12], LPIN1 and FADS2 are involved in the control of fatty acid metabolism at different levels [28, 58], and MYO6 mediates endocytosis within intracellular organelles [23].

**Fig. 3:**
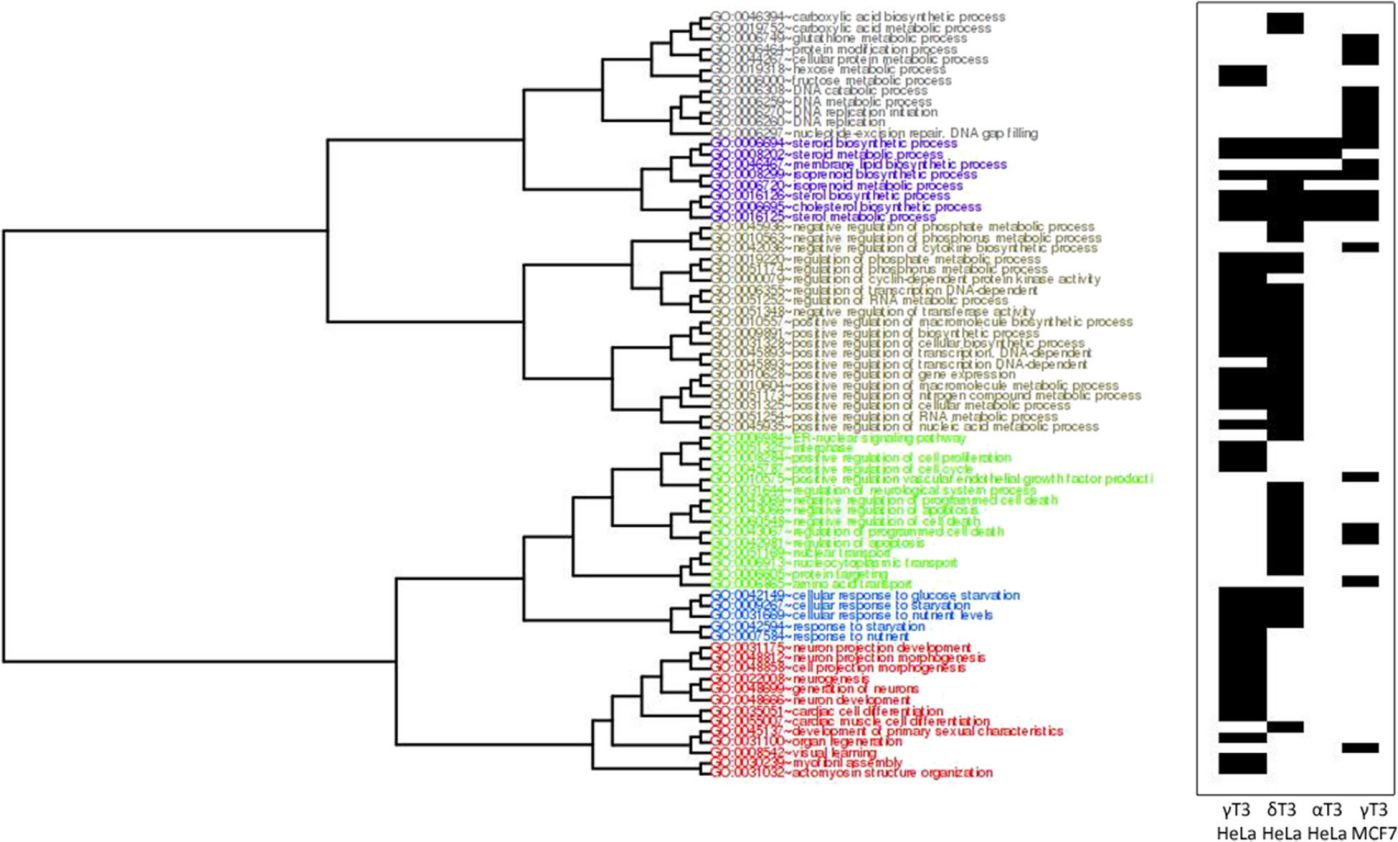
Clustering analysis of biological processes modulated by T3 treatment. Biological processes enriched by differentially expressed genes in HeLa (24 h) and MCF-7 (24 h) cells treated with T3 are represented in the *plot with black blocks*. The matrix of semantic similarities between each pair of significantly enriched biological processes was estimated by Resnik measure. Hierarchical cluster analysis was performed on the obtained matrix. The optimal cluster division was estimated by the analysis of silhouette scores. Each cluster is represented by *different colors* in the dendrogram

In order to identify ERβ-independent pathways of apoptosis activation, we compared γ-T3-related changes in gene expression profiles, previously observed in MCF-7 with those obtained in HeLa. Figure 3 shows that the effect of T3 treatment only partially overlaps in the two cell lines. In fact, in both cell lines, we observed a significant modulation of isoprenoid metabolism and sterol and steroid biosynthetic processes. In HeLa cells, we also observed an enrichment of biological processes related to the regulation of the cell cycle, hexose, and carboxylic acid metabolism. Similarly, we observed an enrichment of the biological processes related to the regulation of apoptosis, cytokines biosynthesis, and glutathione metabolism associated with the profiles of genes differentially expressed in MCF-7. Several evidences indicated that these processes have a role in regulating a common phenotype related to cellular death [7, 13].

Conversely, the profile of differentially expressed genes in HeLa cells specifically involved processes related to tissue development and regulation of transcription, suggesting the activation of specific molecular mechanisms distinct from those activated in MCF-7.

A similar functional analysis was performed to identify cellular compartments annotated in GO and mainly enriched by the profiles resulting from gene modulation affected by T3 treatment.

In HeLa, the treatment with γ-T3 was mainly associated with the modulation of gene products located within intracellular organelles and in particular at the level of the EndoR. The same cellular compartment was identified as a target also in our previous experiments only focusing on MCF-7 cells (Additional file 2: Figure S1). However, in MCF-7, γ-T3 treatment mainly resulted in the modulation of profiles related to mitochondria and Golgi apparatus.

In both MCF-7 and HeLa cells, γ-T3 treatment induced a significant modulation of sterol and steroid biosynthetic processes and of processes related to isoprenoid metabolism.

In order to investigate the relationship between “death-committed” phenotype and the involvement of EndoR identified by the analysis of GO cellular compartment enrichment, we performed a more specific analysis, to further characterize the gene expression profile induced by T3 treatments and specifically related to EndoR stress.

Utilizing a data-mining approach, we built a list of 568 genes, for which evidences exist in the literature that indicate an involvement in EndoR stress. This list was utilized to map the profiles of differentially expressed genes observed in HeLa experiments. This approach suggests that γ-T3 and δ-T3 have a stronger ability to modulate the expression of genes related to EndoR stress with respect to α-T3. Only three genes related to EndoR stress were downregulated by all T3 (SREBF1, SCD, LPIN1). Both γ-T3 and δ-T3 downregulated three genes (SREBF2, CDKN1A, ID2) and upregulated four genes (HSPA5, ASNS, PHLDA1, GDF15). γ-T3 specifically upregulated seven genes (CCND1, CHAC1, DNAJB9, FAS, GEM, GFPT1, XBP-1), whereas δ-T3 downregulated three genes (GSK3B, DNAJC10, JUN) and upregulated four genes (SDF2L1, BCR, TRIB3, FAM129A). Noteworthy, several genes belonging to this group are established markers of EndoR stress (ASNS, CDKN1A, FAS, XBP-1, SDF2L1, TRIB3, HSPA5) [2, 19, 24, 29, 36, 63].

### T3-induced Ca^2+^ release in EndoR

T3-induced changes in gene expression profile that were obtained by microarray experiments, eventually confirmed by RT-PCR (data not shown), suggested the presence of EndoR stress upstream to the apoptosis induced by T3. We therefore tested the occurrence of one of the first, more evident signals associated with EndoR stress: the intracellular Ca^2+^ release from the endoplasmic reticulum.

**Fig. 4:**
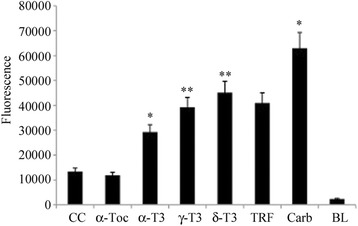
T3-induced Ca^2+^ release in the cytosol. Quantitation of Ca^2+^ release by Fluo-4 NW Calcium Assay. HeLa cells were incubated in the presence of α-TOC, α-T3, γ-T3 or δ-T3, and TRF (10 μg/ml) for 15 min. As the positive control, cells were treated with 50 μM carbachol. *CC* indicates treatment with the vehicle only (DMSO)

Figure 4 shows that α-T3, γ-T3, and δ-T3, but not TRF, induced a marked and significant Ca^2+^ release from the endoplasmic reticulum to the cytoplasm, confirming the involvement of EndoR stress in T3-induced apoptosis. It is interesting to note that α-TOC had no significant effect on Ca^2+^ release, further indicating that the observed effects are definitively structure-specific.

### Characterization of T3-induced EndoR stress

**Fig. 5:**
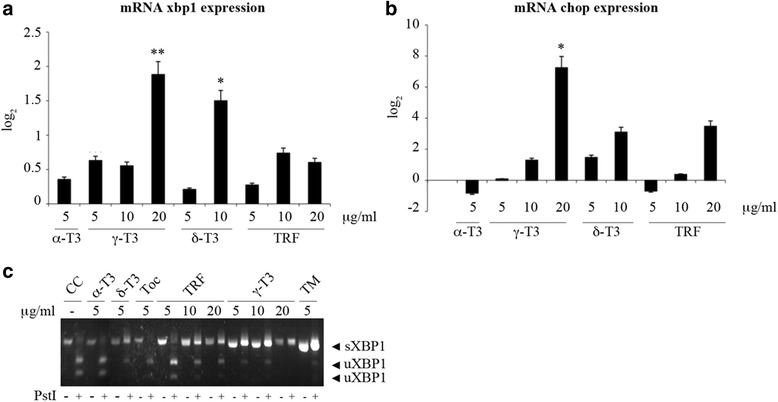
γ-T3- and δ-T3-induced expression of EndoR stress genes after 24 h of treatment. **a** Effect of γ- and δ-T3 and α-T3 on XBP-1-mRNA expression. γ-T3 (20 μg/ml) and δ-T3 (10 μg/ml) induce significant mRNA expression of XBP-1. **b** Effect of γ- and δ-T3 and α-T3 on chop-mRNA expression. Only γ-T3 (20 μg/ml) induces significant mRNA expression of chop. **c** α-T3 (5 μg/ml), δ-T3 (5 μg/ml), TRF (5–20 μg/ml), and γ-T3 (5–20 μg/ml), but not α-TOC (5 μg/ml), induce the alternative splicing of XBP-1. Details about the Pst1 digestion are provided in the “Methods” section. Tunicamycin (TM) was used as a positive control of EndoR stress induction. *Asterisks* indicate significant differences (*p* value ≤0.05) between treated cells vs control (*CC*)

X-box binding protein-1 (XBP-1) and C/EBP homology protein (CHOP) are two of the major players involved in EndoR stress response. At 24 h from the administration of γ-T3 (20 μg/ml) and δ-T3 (10 μg/ml), we observed an increased expression of total XBP-1. Conversely, CHOP messenger RNA (mRNA) expression only increased in association with the treatment with the highest (20 μg/ml) concentration of γ-T3 (Fig. 5a, b). In agreement with the absence of effect on Ca^2+^ release, the treatment with α-TOC and TRF did not induce any significant changes of XBP-1 and CHOP expression (data not shown).

The proteolitic digestion with Pst1 restriction enzyme allows to discriminate between the unspliced and spliced forms of XBP-1. In fact, the splicing of this mRNA is associated with the loss of Pst1 “cutting” site within XBP-1-mRNA. Therefore, after Pst1 digestion, the unspliced form is visualized as two separate bands (at 291 and 307 kD), while the spliced form is visualized as one band only (at 572 kD), typical of EndoR stress. Figure 5c shows that TRF (10 and 20 μg/ml), γ-T3 (5, 10, and 20 μg/ml), and δ-T3 (5 μg/ml) induced the alternative splicing of XBP-1 (sXBP-1).

Also in this case, α-TOC had a different effect, only inducing the expression of the unspliced form, uXBP-1. Therefore, according to the notion that XBP-1 alternative splicing is mediated by inositol requiring enzyme-1α (IRE-1α), we hypothesized that γ- and δ-T3 treatment indirectly modulates IRE-1α activity.

### The treatment with tocotrienols did not induce PERK phosphorylation and ATF-6 cleavage but only activate the IRE-1α pathway

At least three different molecular pathways have been reported to be affected by EndoR stress [5]. Therefore, together with the expression of IRE-1α, we investigated the expression of protein kinase RNA (PKR)-like ER kinase (PERK) and of the activating transcription factor-6 (ATF-6). To this aim, we selected the concentrations of α-, γ-, and δ-T3 that were associated with a more evident effect on gene expression. As positive control for EndoR stress induction, HeLa cells were treated with 5 μg/ml tunicamycin (TM).

**Fig. 6:**
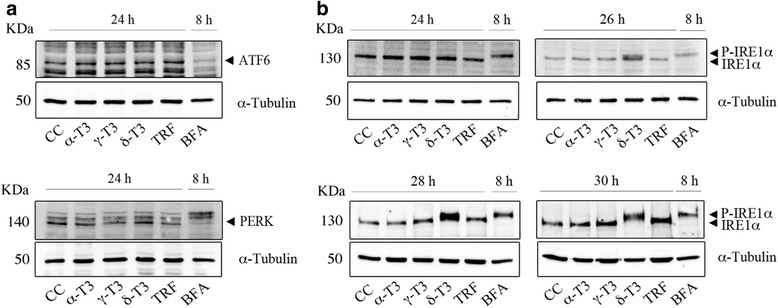
Effects of TRF and purified T3 on PERK, ATF-6, and IRE-1α activity. T3 (10 μg/ml) affect the UPR intracellular pathway in HeLa cells. *CC* indicates the treatment with the vehicle only (DMSO). Brefeldin (*BFA*) (2.5 μg/ml) was used as a positive control of EndoR stress. α-Tubulin was used as the loading control. **a** T3 treatment has no effect on the expression of ATF-6 and PERK proteins at 24 h from the treatment. **b** IRE-1 protein expression and phosphorylation are significantly affected by T3 treatment. The figure shows one representative experiment out of at least three separate experiments. Densitometry and statistical analysis is showed as Additional file 3: Figure S2

Figure 6a (and Additional file 3: Figure S2) shows that any of T3 (10 μg/ml) induced either ATF-6 proteolytic cleavage or PERK phosphorylation at 24 h and at longer incubation times (not shown). To better characterize the effect of T3 on the IRE-1α phosphorylation, we performed a time-course observation in HeLa cells incubated with 10 μg/ml of α-, δ-, and γ-T3 and TRF at different time points (24, 26, 28, and 30 h). At 26 h incubation, only δ-T3 treatment was associated with a shift of the electrophoretic mobility of IRE-1α (Fig. 6b and Additional file 3: Figure S2) in comparison to control (lane cc), indicating the occurrence of protein phosphorylation and, therefore, the activation of this pathway. In addition, longer exposures to δ-T3 (28 and 30 h) were associated with IRE-1α phosphorylation, indicating the presence of a long-term maintenance of protein activation only upon the treatment with δ-T3.

### Effects of TRF and purified T3 on caspase activity

**Fig. 7:**
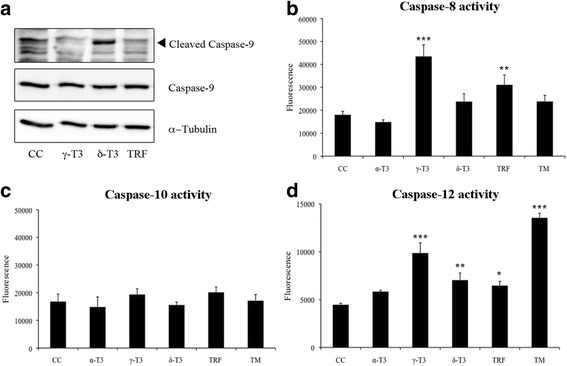
Effect of T3 on caspase activity. T3 modulate caspase-9 protein levels (**a**) and caspase-8, caspase-10, and caspase-12 activity (**b, c**, and **d**, respectively) in HeLa cells**.** Cells were treated with α-T3, γ-T3, δ-T3, or TRF (10 μg/ml) for 12 h or TM (5 μg/ml) for 4 h; *CC* indicates treatment with the vehicle only (DMSO). The *panels* show one representative experiment out of at least three separate experiments. The *asterisks* indicate significant differences (*p* value ≤0.05) between treated cells vs control (*CC*)

It is known that one of the outcomes of EndoR stress is the activation of apoptosis [11]. Twelve hours after the administration of γ-T3 or TRF (10 μg/ml), we detected a significant increase of caspase-12 and caspase-8 activity (Fig. 7b, d). Moreover, δ-T3 treatment (10 μg/ml) induced a modest but significant increase of caspase-12 activity. On the contrary, caspase-10 activity was insensitive to all the different treatments (Fig. 7c). Finally, we observed caspase-9 cleavage only in association with δ-T3 administration (Fig. 7a).

### Effects of PBA on GRP78 protein expression and caspase-12 activity

Glucose-regulated protein 78 (GRP78) is a natural molecular chaperone. It is expressed as a response to EndoR stress, and it is involved in protein folding, proteasome degradation, EndoR Ca^2+^ binding, and also in the control and activation of transmembrane EndoR stress sensors. GRP78 is therefore one of the major upstream regulators of ER protein folding, directly involved in the activation of ATF-6, IRE-1, and PERK.

In order to assess the real involvement of EndoR stress in the apoptosis induced by T3, we measured the GRP78 protein level in the presence of a chemical chaperone, sodium 4-phenylbutyrate (4-PBA), that stabilizes protein structure improving cellular folding capacity, therefore reducing EndoR stress.

Firstly, we performed a dose-response curve to evaluate the appropriate 4-PBA concentration. HeLa cells were incubated with different concentrations of 4-PBA (5, 8, 10 mM) with or without TM for 4 h. Then, we utilized the lowest 4-PBA concentration (5 mM) not associated with toxic effect on HeLa proliferation (data not shown).

**Fig. 8:**
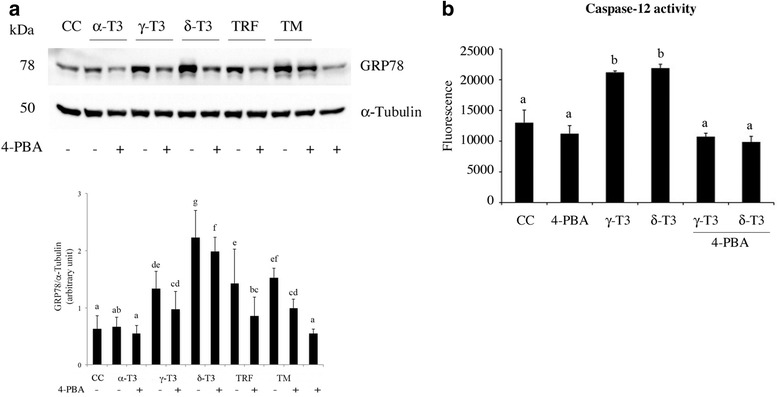
Effects of PBA ER stress inhibitor on GRP78 protein levels and caspase-12 activity. T3 modulates the expression level of EndoR molecular chaperone GRP78. HeLa cells were treated with α-T3, γ-T3, δ-T3, or TRF (10 μg/ml) for 24 h. *CC* indicates the treatment with the vehicle only (DMSO). Tunicamycin (*TM*) (5 μg/ml, for 4 h) was used as a positive control of EndoR stress induction. 4-PBA was used as the specific inhibitor of EndoR stress. α-Tubulin was utilized as a loading control. **a** GRP78 protein levels are affected by T3 treatment, and the co-treatment with 4-PBA (5 mM) prevents the changes induced by T3. The figures show one representative experiment out of at least three separate experiments. Data obtained by densitometry were log_2_ transformed in order to obtain data symmetrically distributed and were analyzed by one-way ANOVA with repeated measures followed by Fisher’s test. *p* values ≤0.05 were considered to be statistically significant. *Different letters* indicate significant differences between groups (p < 0.05). **b** co-treatment with 4-PBA after 24 h significantly inhibits γ-T3- and δ-T3-induced increase of caspase-12 activity. Data were analyzed by one-way ANOVA with repeated measures followed by Fisher’s test. *p* values ≤0.05 were considered to be statistically significant. *Different letters* indicate significant differences between groups (*p* < 0.05)

HeLa cells were treated with T3 and TRF (10 μg/ml) and co-incubated with or without 4-PBA (5 mM) for 24 h. Figure 8a shows that the treatment with δ- and γ-T3, and TRF is associated with an increase of GRP78 protein level. δ-T3 had a more remarkable effect in comparison with other T3 forms and TRF. 4-PBA significantly reduced GRP78 increase induced by δ-T3 and TRF treatment.

Finally, we assessed the effect of T3 on caspase-12 activity, which is the final effector of EndoR stress-induced apoptosis. Figure 8b shows that, after 24 h, co-incubation with 4-PBA significantly reduced the activity of caspase-12 with respect to γ- and δ-T3 treatment.

### XBP-1 activation in MCF-7 breast cancer cells not expressing ERβ

To further confirm that T3-induced XBP-1 alternative splicing was independent of the presence of ERβ, we repeated the experiment on a specific clone of MCF-7 breast cancer cells not expressing ERβ [8].

Similar to HeLa, the administration of γ-T3 (20 μg/ml) and δ-T3 (10 μg/ml) to a clone of MCF-7 cells not expressing ERβ induced the alternative splicing of XBP-1 (sXBP-1). Also in this case, α-T3 induced only the expression of the unspliced form, uXBP-1.

## Discussion

Several studies have shown that T3 inhibit cell proliferation and induce apoptosis in human and murine breast cancer cells [22, 34, 43, 55] and in many other types of tumor cells [17, 52, 59]. More recently, the beneficial effects of T3 on different human disorders have been evaluated in clinical trials [1, 33, 42, 45]. Within this context, few studies have definitively addressed the molecular mechanisms underlying T3 activity. We have previously demonstrated a novel molecular mechanism involving the interaction of γ- and δ-T3 with estrogen receptor-β [15, 16]. In these studies, we reported that the activated complex ERβ/T3 translocates into the nucleus to modulate the expression of specific genes related to the apoptotic response and containing ERE in their promoters. However, as mentioned above, the antiproliferative activity of T3 has also been observed in several other cell types, including melanoma, prostate cancer and lung and liver carcinoma, and in particular in HeLa [61], a cell type known to be void of any form of canonical ERs. Therefore, we considered the possibility of an alternative pathway for the modulation of apoptotic response, independent of ER activity.

We conducted a set of preliminary experiments, showing that T3 modulate the expression of a pro-apoptotic gene, containing ERE sequences, also in HeLa cells, where this expression is obviously not driven by the activation of ERs. In particular, we observed a significant increase of MIC-1 and cathepsin D expression in response to the administration of γ- and δ-T3 (data not shown). Starting from this background, we planned and carried out a first “problem-driven” protocol, utilizing a transcriptomic approach to identify the profile of differential gene expression associated with T3 treatment in HeLa cells. A “data-mining process” identified a significant modulation of several genes involved in EndoR stress following the treatment with γ- and δ-T3. According to the similarity between the biological processes enriched in HeLa and MCF-7 following the administration of T3, we focused our attention on their role in activating EndoR stress, a pathway identified in both MCF-7 and HeLa (see Fig. 3).

A wide spectrum of genes related to EndoR was significantly affected by T3. Among them, we observed a downregulation of SCD expression that has been reported to be associated with an increase of EndoR stress in response to palmitate and to apoptosis in pancreatic β-cells [21]. Similarly, we observed a downregulation of the LPIN gene, already reported to be induced by EndoR stress ([37]) and a downregulation of SREBF1 and SREBF2 by γ-T3 and δ-T3. The expression of these genes, in particular SREBF2, is activated within the unfolded protein response (UPR) pathway to cope with EndoR stress [14]. We could therefore conclude that both γ-T3 and δ-T3 affect the ability of UPR to react to stress stimuli. The molecular specificity of this effect is quite high, as several transcription factors related to EndoR stress are modulated by γ- and δ-T3, but not by α-T3. Interestingly, after δ-T3 and γ-T3 treatment, we also observed the upregulation of HSPA5 (or GRP78), a molecular chaperone that promotes protein folding and inhibits protein aggregation in the EndoR [35].

On the basis of the results obtained by microarray, we concluded that specific T3 forms induce apoptosis in HeLa cells via EndoR stress. The precise reason why specific isomers are more effective than others and the molecular mechanisms underlying these distinct effects are still scarcely understood.

EndoR stress has already been identified as one of the major pathways involved in the initiation of apoptosis, and it is known that EndoR stress-induced apoptosis is strictly associated with Ca^2+^ release in cytoplasm [50]. In fact, EndoR acts as an intracellular store and retains Ca^2+^ at a concentration thousands of times higher than those present in the cytosol. Ca^2+^ disposal is modulated by EndoR-located Ca^2+^ channels that, in the presence of EndoR stress, release Ca^2+^ from the lumen to activate specific Ca^2+^-depending signals and, eventually, apoptosis [49]. In the present study, we observed Ca^2+^ release to the cytoplasm of HeLa cells immediately (15 min) after T3 treatment (Fig. 4). This observation led our focus on the three major molecular pathways that characterize EndoR stress.

As mentioned, at least three different transmembrane proteins are involved in EndoR stress, namely the following: PERK, ATF-6, and IRE-1 [49]. These proteins remain inactive and bound to glucose-regulated proteins (GRPs) and, in particular, to GRP78 also known as “immunoglobulin heavy chain-binding protein” (BiP) or HSPA5, a chaperone member of the heat-shock protein-70 (HSP70) family that plays an important role in regulating the UPR pathway. Under condition of EndoR homeostasis, GRP78 remains in its inactive form and constitutively binds to three UPR transmembrane sensors, ATF-6, PERK, and IRE-1 [51]. Following EndoR stress, the complex BiP/EndoR protein is dissociated, released from UPR sensors and activated [11].

**Fig. 9:**
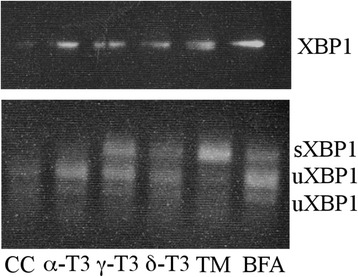
XBP-1 activation in a ERβ silenced clone of breast cancer cells (MCF-7). A specific clone of MCF-7 cells, not expressing ERβ, was incubated with δ-T3 (20 μg/ml), γ-T3 (10 μg/ml), α-TOC, and α-T3 (20 μg/ml). Details about Pst1 digestion are provided in the “Methods” section. *CC* indicates treatment with the vehicle only (DMSO). Brefeldin (*BFA*) was used as a positive control of EndoR stress induction. The figure shows one representative experiment out of at least three separate experiments

As shown in Fig. 6, treatment with T3 (γ-T3, δ-T3, and TRF) did not induce a differential expression of proteins PERK and ATF-6, while the expression of IRE-1α phosphorylation was significantly modulated. In fact, at 24 h from T3 treatment, IRE-1α phosphorylation was faintly detectable, while the splicing of XBP-1 pre-mRNA, a downstream step to IRE-1 activity, was evident and detectable (Fig. 5c) both at shorter and longer exposure times (12 and 48 h, data not shown). We confirmed that the pathway leading to the expression of a spliced form of XBP-1, induced by γ- and δ-T3, is not cell-specific and ERβ-dependent by utilizing a specific clone of MCF-7 that has been reported to be void of ERβ [8]. Similarly, to HeLa cells, the administration of γ- and δ-T3, but not α-T3, to this cell line was associated with the expression of a truncated form of XBP-1 (see Fig. 9). The apparent discrepancy between IRE-1α phosphorylation and XBP-1 splicing at 24 h from T3 treatment could be explained by the lesser sensitivity of western blot methodology in comparison with PCR assay.

We observed an evident variability of IRE-1 phosphorylation suggesting that, in our experimental conditions, this protein is likely to oscillate between two different states, either “active” or “refractive” while maintaining its endoribonuclease activity. This interpretation is supported by a study published by Li and coworkers [31] that described a *three-state* model for the activation of mammalian IRE-1α: (i) an inactive state that can be “switched on” by EndoR stress; (ii) an active state characterized by IRE-1α oligomerization and XBP-1 splicing; and (iii) a refractive state in which IRE-1α enters after a prolonged activation, no longer responding to an “unresolved” EndoR stress. The same authors reported that different IRE-1α forms are not necessarily recognized by the anti-p-IRE-α antibody and suggested that de-phosphorylation has an important role for the entering into the refractive state. Other authors [25] demonstrated that, upon persistent EndoR stress (e.g., inositol depletion), IRE-1 is weakly but continuously activated in a non-clustered form through its association with BiP. The same study reports that the weak activity of IRE-1α might act as an indispensable “fine tuning” for cell adaption to chronic EndoR stress conditions. When homeostasis is not recovered, UPR signaling induces cell death by apoptosis.

Besides affecting IRE-1, we observed that treatment with T3 is associated with a significant modulation of XBP-1 and CHOP mRNA expression, starting at 24 h from administration (Fig. 5a, b).

Overall, our data indicate that specific forms of T3 are able to induce EndoR stress in HeLa cells through the activation of IRE-1α (fluctuating between active/refractive forms), which in turn mediates the alternative splicing of XBP-1 mRNA, and modulates CHOP transcription. A prolonged EndoR stress leads to an increase of CHOP expression that switches EndoR stress signaling from “pro-survival” to “pro-apoptosis” [56, 62]. We confirmed our hypothesis utilizing the EndoR stress inhibitor, 4-PBA. In fact, co-treatment with 4-PBA significantly reduced both GRP78 protein levels and caspase activity induced by γ- and δ-T3.

Finally, if cells undergoing EndoR stress do not succeed in restoring cellular homeostasis and degrade protein aggregates, UPR leads to cell cycle arrest and, subsequently, to apoptosis [48]. In other words, in our experimental conditions, T3 treatment “forced” HeLa cells toward apoptosis.

Accordingly, we have shown that specific forms of T3 activate caspase-12 and caspase-8 expression (see Fig. 7). Although T3 treatment has already been reported to activate EndoR stress [44, 60], no studies had previously addressed the role of these molecules in inducing EndoR-mediated apoptosis in a model different from breast cancer. It is interesting to note that caspase-12, an EndoR resident caspase, is specifically cleaved and activated during EndoR stress, but not following the activation of death receptors and other mitochondria-related apoptotic signals. Ca^2+^ efflux from EndoR during stress activates calpain that, in turn, activates EndoR-caspase-12 [39]. Once activated, caspase-12 can trigger the maturation cascade finally leading to the activation of caspase-3 to complete the apoptotic program [38]. In HeLa cells, we observed the activation of caspase-12 both following γ- and δ-T3 treatment, but only the latter was associated with caspase-9 cleavage (Fig. 7). Even though we could not observe any direct evidence of IRE-1α phosphorylation associated with γ-T3 treatment, the presence of evident downstream consequences to its activation lets us hypothesize that, at least in HeLa cells, also γ-T3 is involved in the UPR pathway.

In our study, we also observed a significant activation of caspase-8, even though this protease is known to play a role in death receptor-mediated apoptosis [47]. Therefore, we cannot exclude a possible involvement of a death receptor-related pathway within the spectrum of T3 activities.

**Fig. 10:**
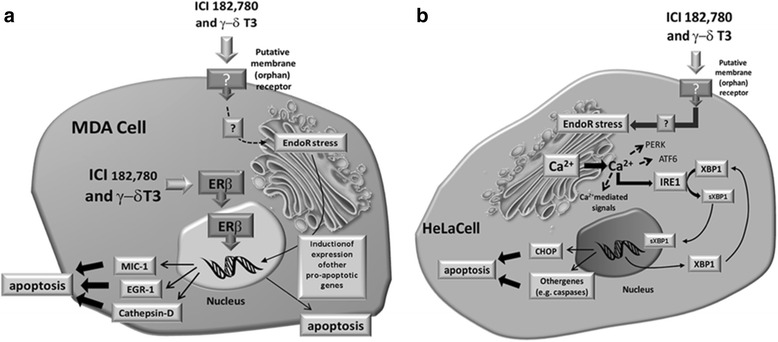
**a** In tumor cells expressing ERβ (MDA-MB-231, MCF-7), γ- and δ-T3 bind to and activate the ERβ receptor, inducing the expression of pro-apoptotic genes such as MIC-1, EGR-1, and cathepsin-D, finally triggering apoptosis. More details about this mechanism are described in (6). Also in these cell types, a contribution by pro-apoptotic signals originating from EndoR is not to be excluded. **b** In tumor cells not expressing any of the canonical forms of ERs (ERα and ERβ), γ- and δ-T3 induce EndoR stress activating Ca^2+^ release in the cytoplasm. Overall, the treatment with specific forms of T3, but not α-TOC, is associated with specific Ca-dependent signals involved in the unfolded protein response (UPR), the core pathway to cope with EndoR stress in eukaryotic cells. In particular, Ca^2+^ release is followed by the activation of IRE-1, which in turn activates XBP-1 splicing, the binding of this latter to DNA, and the subsequent induction of the expression of pro-apoptotic genes. The expression of other genes associated with EndoR stress (PERK and ATF-6) is not significantly affected by T3. Our data also provide ground to speculate about the presence of a putative (orphan) receptor, possibly located at the level of the cellular membrane able to bind T3 and other estrogen mimetics such as ICI-182,730. This mechanism could be active in several cell types, independently of the presence of functional ERs

Our data provide ground to speculate about the presence of a putative (orphan) receptor, possibly located at the level of the cellular membrane able to bind T3 and other estrogen mimetics such as ICI-182,730. This mechanism could be active in several cell types, independently of the presence of functional ERs. In fact, previous (unpublished) observations from our group have unexpectedly indicated that, also in HeLa, the treatment with the specific ER inhibitor ICI-182,780 weakens the effects of T3 on MIC-1 gene expression (Additional file 4: Figure S3). This evidence allows us to speculate that a hypothetical “specific” (orphan) receptor characterized by a pocket able to also bind ICI-182,780 (see Fig. 10) exists, also able to bind γ- and δ-T3. According to the chemo-physical characteristics of T3, the candidate downstream target(s) of the activity of this receptor could reasonably be located at the level of EndoR. The activation of this hypothetical (orphan) receptor would sequentially trigger EndoR stress, IRE-1 activation, and XBP-1 splicing. Once synthesized, sXBP-1 would modulate the expression of a specific set of genes, inducing apoptosis.

## Conclusions

Our study demonstrates that γ- and δ-T3 activate a series of specific cellular responses leading to apoptosis also in cells lacking of ERβ. We have identified and characterized an EndoR stress-dependent pathway activated by γ- and δ-T3 that is likely to cooperate with ERβ (when present)-dependent signaling in triggering apoptosis in several tumor cell types. Further future studies must address the molecular mechanism by which γ- and δ-T3 induce EndoR stress.

## Methods

### Chemicals

TRF was obtained from Golden Hope Plantation (Malaysia) and purified as previously described [53]. T3 and α-TOC constituted more than 95% of the final TRF composition, the remaining being minor components (carotenoids, flavonoids). TRF typically contained 32% α-TOC, 25% α-T3, 29% γ-T3, and 14% δ-T3.

Purified T3 were provided by Dr. Hiroyuki Yoshimura at Eisai Food and Chemical Co., Ltd (Tokyo, Japan). Purity was ~99% for all T3. Pure α-TOC (≥95.5%) was purchased by Sigma-Aldrich (St. Louis, MO, USA). The real concentration of T3 and α-TOC solutions was determined spectrophotometrically from the specific extinction coefficients (ε_292_ α-TOC = 75.8; ε_292.5_ α-T3 = 91; ε_296_ γ-T3 = 90.5; ε_297_ δ-T3 = 89.1) before each experiment. Stock solutions of TRF and T3 were stored at–20 °C in aliquots and diluted to the desired concentration in dimethyl sulfoxide (DMSO).

### Cells lines and treatments

HeLa cells were obtained from the American Tissue Culture Collection (Manassas, VA, USA). A clone of MCF-7 breast cancer cells, not expressing ER-β [8], was a gift of Prof M. Marino.

Cells were grown in DMEM medium (Euroclone, Pero, Milan, Italy) supplemented with 10% fetal bovine serum (FBS, Sigma-Aldrich), 100 U/ml penicillin and 100 μg/ml streptomycin (Pen/Strep, Euroclone), 2 mM glutamine (Euroclone), and 1% non-essential amino acid (Sigma Aldrich). Cells were maintained at 37 °C in a humidified atmosphere of 5% CO_2_/95% air.

Before any experimental session, cells were synchronized in G_1_/G_0_ by starvation in serum-free medium for 2 days. Once synchronized, 300.000 cells were seeded onto multi-well plates. TRF, purified T3, or α-TOC were dissolved in DMSO and individually added to the culture medium. When not differently indicated in the text, incubation time was 24 or 48 h. The final TRF concentration in culture media was 10 μg/ml. Purified α-TOC and T3 were added to the medium at the following concentrations: α-TOC was 5 μg/ml; α-T3 was 5 μg/ml; γ-T3 was 5, 10, and 20 μg/ml; and δ-T3 was 5 and 10 μg/ml. These concentrations have been selected in order to facilitate the comparison with previous published studies by our group or by others. Control cells were treated with an equal volume of DMSO alone.

Tunicamycin (TM) and brefeldin A (BFA) were utilized as inducers of EndoR stress response. Preliminary investigations indicated that they have identical effects on our cellular model. Treatments with 2.5 μg/ml BFA for 8 h or 5 μg/ml TM for 4 h were therefore utilized as a positive control for EndoR stress. Different concentrations (5, 8, and 10 mM) of sodium 4-phenylbutyrate (4-PBA, Calbiochem, USA), a specific inhibitor of EndoR stress, were administered to HeLa along with T3 or TRF.

### DNA laddering

DNA extraction was performed according to Gooch and Yee [20], and with minor modifications previously described [15], extracted DNA was electrophoresed in 1.5% agarose gels containing ethidium bromide (EtBr) and visualized by UVIpro Bronze acquisition system (UVITEC, Cambridge, UK).

### Calcium release assessment and free calcium assay

Free Ca^2+^ concentration was determined by the Fluo-4 NW Calcium Assay Kit (Invitrogen) according to the manufacturer’s instructions. HeLa cells were incubated in the presence of TRF, α-TOC, α-T3, γ-T3, or δ-T3 (10 μg/ml), and at the end of the incubation time, fluorescence was measured (494-nm excitation/516-nm emission) by a Tecan fluorometer (TECAN Infinite® 200 PRO).

### cDNA hybridization and microarray data analysis

Two micrograms of high-quality total RNA were sent to ServiceXS BV (Leiden, The Netherlands) and processed according to the Affymetrix protocol (Affymetrix Eukaryotic One-Cycle Target Labeling and Control reagents) to generate biotin-labeled antisense cRNA (Complementary RNA). Labeled cRNA was hybridized to the NuGO Affymetrix Human Genechip NuGO_Hs1a520180 (custom designed by the European Nutrigenomics Organization NuGO, consisting of 23,941 probe sets including 71 control probe sets).

Cell intensity files (*.cel) for each GeneChip processed were generated using Command Console Software.

Three biological replicates were generated for each experimental condition. Microarray statistical analysis was performed using oneChannelGUI R package.

Raw signal intensities were normalized using GCRMA method as background correction, and differentially expressed genes were identified with LIMMA analysis selecting only the genes with a fold change (T3 treatment vs control) of at least 0.5 and a *p* value threshold above 0.05. The analysis of differential expression by the LIMMA method is based on an empirical Bayesian approach and has been reported to be reliable also in the case of small and unequal sample sizes (ref: PMID:16646809).

Specific up- and downregulated genes from microarray analysis were selected for a technical validation by RT-qPCR technique.

### Pathway and network analysis

The set of modulated genes identified by microarrays was submitted for the analysis of enrichment of gene ontology (GO) biological processes and cellular components (level 5) utilizing the DAVID web server [18]. Significant enriched GO biological processes were identified according to a *p* value <0.05.

Overrepresented biological processes and cellular components modulated by γ-T3 in HeLa and MCF-7 were compared utilizing the GOSim package on the basis of the Resnik method to assess their semantic similarities. The obtained matrix of similarity distances was clustered by the Ward method, and the optimal number of clusters used to identify the groups of similar GO terms was assessed choosing the number of clusters that produces the best silhouette score. Similar analysis was performed to compare overrepresented terms according to the treatment with different T3 in HeLa cells.

A specific list of genes related to EndoR stress was built by merging the annotation obtained from the UniProt database and PubMed. The search on PubMed was performed by the Agilent literature search of Cytoscape software [32], searching about 1000 papers having “endoplasmic reticulum stress” and “Homo sapiens” within the keywords.

### RNA isolation and real-time PCR measurements

Total RNA and real-time PCR analysis have been performed as previously described in the detail [15]. Primers used in real-time PCR measurements (see Table 1) have been designed according to the available literature of by a specific software (Primer Express™). Quantitative differences in cDNA target among samples were measured according to the mathematical model of Pfaffl [46]. The expression ratio was determined for each sample by calculating (*E*_sample_)^ΔCt(sample)^/(*E*_control_)^ΔCt(control)^, where *E* is the efficiency of the primer set and ΔCt = Ct_(control)_ − Ct_(sample)_. Different normalization options, based on a set of different “housekeeping” genes, have been tested (data not shown) and provided no significant differences at the level of differential gene expression detection. β-actin was therefore selected to normalize expression data. Finally, results have been log_2_ transformed in order to obtain symmetrically distributed data. The amplification efficiency of each primer set was calculated from the slope of a standard amplification curve of log μl cDNA/reaction vs Ct value over at least 4 orders of magnitude (*E* = 10^−(1/slope)^); β-actin primers, *E* = 2.15; MIC primers, *E* = 2.03; cathepsin D primers, *E* = 2.55; CHOP primers, *E* = 1.94; XBP-1 primers, *E* = 2.19.

**Table 1:** List of genes considered on the basis of data interrogation and primers utilized in RT-PCR and PCR assay

Gene	GenBank	Primers 5′→3′	Size template
β-actin (RT-PCR)	NM_001101.3	F: AGAAGGATTCCTATGTGGGGG	101 bp
		R: CATGTCGTCCCAGTTGGTGAC	
MIC-1 (RT-PCR)	NM_004864	F: TGGTGCTCATTCAAAAGACCG	123 bp
		R: GTGGAAGGACCAGGACTGCTC	
Cathepsin D (RT-PCR)	NM_001909	F: CTGTGAGGCCATTGTGGACAC	140 bp
		R: CAGCTTGTAGCCTTTGCCTCC	
XBP-1 (RT-PCR)	NM_005080.3	F: GGAGTTAAGACAGCGCTTGGGGA	118 bp
		R: TGTTCTGGAGGGGTGACAACTGGG	
CHOP (RT-PCR)	NM_001195057.1	F: CCACACCTGAAAGCAGACTGATCCA	102 bp
		R: TCATACCAGGCTTCCAGCTCCCA	
hXBP-1 (PCR)	NM_001079539.1	F: AAACAGAGCAGCAGTCCAGACTGC	472(u)bp 448(s)bp
		R: TCCTTCTGGGTAGACCTCTGGGAG	

### RT-PCR analysis of XBP-1 splicing

cDNA was synthesized from total RNA using One-Step RT-PCR SuperScript III reverse transcriptase (Invitrogen) according to manufacturer’s protocol. The primers used for PCR (see Table 1) were specific for human sXBP-1 (hsXBP-1). The PCR conditions were as follows: 95 °C for 5 min, 95 °C for 1 min, 58 °C for 30 s, 72 °C for 30 s, and 72 °C for 5 min, with 35 cycles of amplification. A 289-bp amplicon was generated from unspliced XBP-1 (uXBP-1) and a 263-bp amplicon from spliced XBP-1 (sXBP-1)*.* PCR amplicons were digested by Pst1. The Pst1 cleavage site is located in the 26-nt intron of uXBP-1, which allows differentiation between the uXBP-1 amplicon (cut PCR product) and sXBP-1. Digested and not-digested PCR products were resolved on 2% agarose gels, stained with EtBr. The PCR fragments were visualized with UVIpro Bronze Imaging System (UVitec, Cambridge, UK).

### Protein extraction and western blot

Total protein were extracted as described according to standardized protocols. The antibody considered were the following: XBP-1 (1:5000, anti-mouse, Santa Cruz Biotechnology), PERK (1:1000, anti-rabbit, Cell Signaling Technology), IRE-1α and phospho-IRE-1α (1:1000, anti-rabbit, Cell Signaling Technology), ATF-6 (1:1000, anti-rabbit, Abcam), caspase-9 and cleaved caspase-9 (1:1000, anti-rabbit, Cell Signaling Technology), α-tubulin (1:10,000, anti-mouse, MP Biomedicals), and GRP78 (1:1000, anti-rabbit, Cell Signaling Technology).

In order to detect IRE-1α and phospho-IRE-1α (p-IRE-1α) protein levels, we carried out a cytosolic and nuclear protein extraction as described by Canali et al. [10]. Protein concentration was determined using a commercial assay kit (Bio-Rad Laboratories, Hercules, CA, USA). Protein samples (30 μg per lane) were loaded onto a SDS polyacrylamide gel, and then, proteins were transferred to a polyvinylidene difluoride (PVDF) membrane (Millipore Corp., Bedford, MA, USA). The PVDF membrane was incubated overnight at 4 °C with an appropriate concentration of specific primary antibody. After washing and incubation with 1:2000 goat anti-mouse or goat anti-rabbit peroxidase-conjugated secondary antibodies (Santa Cruz Biotechnology), specific bands were detected by chemiluminescence reagent ECL Plus (Amersham Pharmacia Biotech, Piscataway, NJ) and visualized by Image Quant LAS 4000 (GE Healthcare Life Sciences).

The band analysis tools of ImageQuant TL software (GE Healthcare Life Sciences) were used to select and determine the background-subtracted density of the bands in all the western blot gels.

### Caspase assay

The activity of caspase-8, caspase-10, and caspase-12 was assessed by means of a Biovision kit (Biovision, Lyon, France) according to the manufacturer’s instructions. The assay is based on the detection of cleavage of substrate IETD-AFC (AFC: 7-amino-4-trifluoromethyl coumarin) that emits blue light (*λ*max = 400 nm), upon cleavage of the substrate by FLICE or related caspases, which can be quantified using a fluorometer or a fluorecence microtiter plate reader. Samples were transferred into a 96-well plate, and the fluorescence assessed using a Tecan fluorometer (TECAN Infinite® 200 PRO).

### Statistical analysis and data presentation

Statistical analysis was performed with R software from the R Foundation for Statistical Computing (Vienna, Austria). Data were analyzed by one-way ANOVA with repeated measures followed by Tukey’s test or Fisher’s test. *p* values ≤0.05 were considered to be statistically significant.

Data obtained by transcriptomic platform have been handled according to the MIAME guidelines (see http://www.ncbi.nlm.nih.gov/geo/info/MIAME.html). Raw data have been deposited into the Gene Expression Omnibus (GEO: http://www.ncbi.nlm.nih.gov/geo/query/acc.cgi?acc=GSE48668) publicly accessible database.

Data obtained by real-time PCR have been handled according to the MIQE guidelines [9].

Figures show one out of at least three independent experiments providing similar results. Histograms present the mean (±S.E.) of at least three experiments.

## Additional files

Additional file 1: Table S1. List of genes modulated by T3 according to microarray analysis. Expression levels are represented as log_2_ fold changes. The last column on the right indicates if the gene has been reported to be involved in EndoR stress. (DOCX 49 kb)
Additional file 2: Figure S1. Clusterization of CC enriched after T3 treatment in HeLa and MCF-7 cells. The distances between enriched CC were estimated according to the *Resnik measure*. The optimal number of clusters was estimated by silhouette scores and represented with different colors within the dendrogram. The box on the right side of the figure shows CCs enriched by a specific treatment. (TIF 3584 kb)
Additional file 3: Figure S2. Densitometric analysis of ATF-6, PERK, and IRE-1α activity. Treatments with T3 have no significant effect on the protein expression of ATF-6 and PERK at 24 h from the treatment. Conversely, IRE-1 phosphorylation is significantly affected by T3 treatment. Data were analyzed by one-way ANOVA with repeated measures followed by Fisher’s test. Asterisks indicate significant differences (*p* value ≤0.05) between treated cells vs control (CC). (TIF 13186 kb)
Additional file 4: Figure S3. Modulation of MIC-1 mRNA expression. MIC-1 mRNA upregulation induced by T3 was partially prevented by pre-treatment with ICI-182,780. Once synchronized, 300.000 cells were seeded onto multi-well plates and pre-incubated with ICI-182.780 (10^−5^ M) for 30 min. TRF, purified T3 or α-TOC were added to the culture medium for 24 h. Gene expression was analyzed by real-time quantitative PCR, and results were log transformed (logarithm 2) in order to obtain data symmetrically distributed [46]. Statistical significance was calculated by one-way ANOVA with repeated measures followed by Tukey’s test using ΔCt value. Asterisks (*) indicate significant differences (*p* value ≤0.05) with respect to control. Data represent the pooled values of at least three independent experiments. (TIF 15116 kb)

## Abbreviations

ASNS: Asparagine synthetase (glutamine-hydrolyzing)
BCR: Breakpoint cluster region
CCND1: Cyclin D1
CDKN1A: Cyclin-dependent kinase inhibitor 1A
CHAC1: ChaC, cation transport regulator homolog 1
DHCR24: 24-dehydrocholesterol reductase
DNAJB9: DnaJ (Hsp40) homolog, subfamily B, member 9
DNAJC10: DnaJ (Hsp40) homolog, subfamily C, member 10
FADS2: Fatty acid desaturase 2
FAM129A: Family with sequence similarity 129, member A
FAS: Fas (TNF receptor superfamily member 6)
FDFT1: Farnesyl-diphosphate farnesyltransferase 1
GDF15: Growth differentiation factor 15
GEM: GTP binding protein overexpressed in skeletal muscle
GFPT1: Glutamine-fructose-6-phosphate transaminase 1
GSK3B: Glycogen synthase kinase 3 beta
HSPA5: Heat-shock 70 kDa protein 5
ID2: Inhibitor of DNA binding 2
IDI1: Isopentenyl-diphosphate delta isomerase 1
INSIG1: Insulin-induced gene 1
JUN: Jun proto-oncogene
KLF7: Kruppel-like factor 7
LDLR: Low-density lipoprotein receptor
MIC-1: Macrophage inhibitory cytokine
MMAB: Methylmalonic aciduria (cobalamin deficiency) cblB type
MYO6: Myosin VI
PHLDA1: Pleckstrin homology-like domain, family A, member 1
SCD: Stearoyl-CoA desaturase (delta-9-desaturase)
SDF2L1: Stromal cell-derived factor 2-like 1
SREBF1: Sterol regulatory element binding transcription factor 1
SREBF2: Sterol regulatory element binding transcription factor 2
TRIB3: Tribbles pseudokinase 3
