# Supplementary material for: Tocotrienols induce endoplasmic reticulum stress and apoptosis in cervical cancer cells
Source: Genes Nutr. 2016 Dec 23;11:32. doi: 10.1186/s12263-016-0543-1 (PMC5180413; doi:10.1186/s12263-016-0543-1)
Supplement: Additional file 1: Table S1. — List of genes modulated by T3 according to microarray analysis. Expression levels are represented as log2 fold changes. The last column on the right indicates if the gene has been reported to be involved in EndoR stress. (DOCX 49 kb) [file 12263_2016_543_MOESM1_ESM.docx]

| **Probeset**  **Table 1**. List of genes modulated by T3s according to microarray analysis. Expression levels are represented as log2 fold changes. The last column on the right indicates if the gene has been reported to be involved in EndoR stress. | **Gene**  **Number** | **Official gene symbol** | **Alpha**  **T3** | **Delta**  **T3** | **Gamma T3** | **ER stress involvment** |
| --- | --- | --- | --- | --- | --- | --- |
| 202308_at | 6720 | **SREBF1** | -0,99 | -1,44 | -2,15 | Y |
| 200832_s_at;  223839_s_at | 6319 | **SCD** | -0,89 | -0,72 | -1,12 | Y |
| 212276_at | 23175 | **LPIN1** | -0,87 | -0,76 | -0,81 | Y |
| 201247_at | 6721 | **SREBF2** |  | -0,70 | -0,95 | Y |
| 202284_s_at | 1026 | **CDKN1A** |  | -0,65 | -0,79 | Y |
| 201565_s_at | 3398 | **ID2** |  | -0,61 | -0,56 | Y |
| 230031_at | 3309 | **HSPA5** |  | 0,93 | 0,59 | Y |
| 205047_s_at | 440 | **ASNS** |  | 1,37 | 0,79 | Y |
| 217996_at | 22822 | **PHLDA1** |  | 0,50 | 0,96 | Y |
| 221577_x_at | 9518 | **GDF15** |  | 1,04 | 1,10 | Y |
| 242336_at | 2932 | **GSK3B** |  | -0,59 |  | Y |
| 229588_at | 54431 | **DNAJC10** |  | -0,57 |  | Y |
| 201466_s_at | 3725 | **JUN** |  | -0,53 |  | Y |
| 218681_s_at | 23753 | **SDF2L1** |  | 0,53 |  | Y |
| 226602_s_at | 613 | **BCR** |  | 0,57 |  | Y |
| 218145_at | 57761 | **TRIB3** |  | 0,61 |  | Y |
| 217967_s_at | 116496 | **FAM129A** |  | 0,88 |  | Y |
| 208712_at | 595 | **CCND1** |  |  | 0,55 | Y |
| 219270_at | 79094 | **CHAC1** |  |  | 0,76 | Y |
| 202843_at | 4189 | **DNAJB9** |  |  | 0,62 | Y |
| 215719_x_at | 355 | **FAS** |  |  | 0,52 | Y |
| 204472_at | 2669 | **GEM** |  |  | 0,87 | Y |
| 202722_s_at | 2673 | **GFPT1** |  |  | 0,63 | Y |
| 200670_at | 7494 | **XBP1** |  |  | 0,50 | Y |
| 218729_at | 56925 | **LXN** |  | -0,67 |  |  |
| 226269_at | 54332 | **GDAP1** |  |  | -0,61 |  |
| 212186_at | 31 | **ACACA** |  |  | -0,59 |  |
| NuGO_eht0358343_at | 338811 | **FAM19A2** |  | -0,61 | -0,57 |  |
| 203939_at;  1553994_at | 4907 | **NT5E** |  | 1,08 | 1,30 |  |
| 234974_at | 130589 | **GALM** |  |  | -0,50 |  |
| 214462_at | 9306 | **SOCS6** |  |  | 0,56 |  |
| 212805_at | 158471 | **PRUNE2** |  | -0,63 | -0,69 |  |
| 227022_at | 132789 | **GNPDA2** |  | 0,53 |  |  |
| 238419_at | 90102 | **PHLDB2** |  | -0,53 |  |  |
| 202769_at | 901 | **CCNG2** |  |  | -0,62 |  |
| 214436_at | 25827 | **FBXL2** |  |  | -0,64 |  |
| 214453_s_at | 10561 | **IFI44** |  |  | 0,58 |  |
| 223599_at | 117854 | **TRIM6** |  | -0,87 |  |  |
| 212662_at | 5817 | **PVR** |  |  | 0,62 |  |
| 225639_at | 8935 | **SKAP2** |  |  | -0,61 |  |
| 226333_at | 3570 | **IL6R** | 0,50 |  |  |  |
| 205729_at | 9180 | **OSMR** |  |  | 0,59 |  |
| 204864_s_at | 3572 | **IL6ST** |  |  | 0,53 |  |
| 202245_at | 4047 | **LSS** |  | -0,59 | -0,57 |  |
| 204334_at | 8609 | **KLF7** | -0,50 | -0,58 | -0,66 |  |
| 227146_at | 169714 | **QSOX2** |  | 0,55 |  |  |
| 208883_at | 51366 | **UBR5** |  |  | -0,55 |  |
| 1556579_s_at | 116931 | **MED12L** |  |  | -0,53 |  |
| 226408_at | 8463 | **TEAD2** |  |  | -0,53 |  |
| 227475_at | 94234 | **FOXQ1** |  | 0,52 |  |  |
| 206613_s_at | 9015 | **TAF1A** |  |  | 0,53 |  |
| 235683_at | 143686 | **SESN3** |  | -0,77 | -0,51 |  |
| 231786_at | 3209 | **HOXA13** |  | -0,56 |  |  |
| 203348_s_at | 2119 | **ETV5** |  | 0,59 |  |  |
| 241355_at | 55806 | **HR** |  | -0,54 |  |  |
| 1554576_a_at | 2118 | **ETV4** |  | 0,78 | 1,29 |  |
| 214804_at | 2491 | **CENPI** |  | -0,56 |  |  |
| 205619_s_at | 4222 | **MEOX1** |  |  | -0,58 |  |
| 226837_at | 161742 | **SPRED1** |  |  | 0,53 |  |
| 226820_at | 149076 | **ZNF362** |  |  | -0,72 |  |
| 241348_at;222851_at | 55279 | **ZNF654** |  | -0,82 | -0,62 |  |
| 214077_x_at | 4213 | **MEIS3P1** |  |  | 0,51 |  |
| 204014_at | 1846 | **DUSP4** |  |  | 0,78 |  |
| 210002_at | 2627 | **GATA6** |  |  | 0,67 |  |
| 239468_at | 283078 | **MKX** |  |  | 0,56 |  |
| 227261_at | 11278 | **KLF12** |  | -0,54 |  |  |
| NuGO_eht0342066_at | 148398 | **SAMD11** |  |  | -0,72 |  |
| 235470_at | 51691 | **NAA38** |  |  | -0,52 |  |
| 206858_s_at | 3223 | **HOXC6** |  |  | -0,59 |  |
| 231926_at | 58513 | **EPS15L1** |  | 0,52 |  |  |
| 221911_at | 2115 | **ETV1** |  | 0,58 | 0,76 |  |
| NuGO_eht0244601_x_at | 8970 | **HIST1H2BJ** |  | -0,60 |  |  |
| 209230_s_at | 26471 | **NUPR1** |  | 0,98 | 0,54 |  |
| NuGO_eht0334095_at | 388561 | **ZNF761** |  | -0,60 |  |  |
| 238937_at | 147923 | **ZNF420** |  | -0,62 |  |  |
| NuGO_eht0279004_at | 128553 | **TSHZ2** |  |  | -0,55 |  |
| 201694_s_at | 1958 | **EGR1** |  | -0,60 | -0,51 |  |
| 237206_at | 93649 | **MYOCD** |  |  | -0,81 |  |
| 200940_s_at | 473 | **RERE** |  | 0,55 |  |  |
| 201906_s_at | 10217 | **CTDSPL** |  |  | -0,51 |  |
| 225140_at | 51274 | **KLF3** |  |  | 0,55 |  |
| 212948_at | 23125 | **CAMTA2** |  | 0,72 | 0,84 |  |
| 229022_at | 7543 | **ZFX** |  | -0,69 | -0,61 |  |
| 204351_at | 6286 | **S100P** |  | 0,64 |  |  |
| 232879_at | 64784 | **CRTC3** |  | -0,58 | -0,55 |  |
| 224367_at | 84707 | **BEX2** |  | 0,50 |  |  |
| 217853_at | 64759 | **TNS3** |  |  | -0,61 |  |
| 213407_at | 23035 | **PHLPP2** |  |  | -0,64 |  |
| 222016_s_at | 64288 | **ZNF323** |  |  | -0,53 |  |
| 219529_at | 9022 | **CLIC3** |  | -1,23 | -0,90 |  |
| 200632_s_at | 10397 | **NDRG1** |  |  | -0,54 |  |
| 207847_s_at | 4582 | **MUC1** |  | -0,69 |  |  |
| 200878_at | 2034 | **EPAS1** |  | 0,96 |  |  |
| 236313_at | 1030 | **CDKN2B** |  |  | -0,64 |  |
| 212992_at | 113146 | **AHNAK2** |  | -0,62 | -0,90 |  |
| 221011_s_at | 81606 | **LBH** |  |  | 0,69 |  |
| 228697_at | 135114 | **HINT3** |  |  | 1,67 |  |
| 203787_at | 23635 | **SSBP2** |  |  | -0,50 |  |
| 221601_s_at | 9214 | **FAIM3** |  | 0,53 |  |  |
| 213348_at | 1028 | **CDKN1C** |  | -0,68 | -0,94 |  |
| 217892_s_at | 51474 | **LIMA1** |  |  | -0,62 |  |
| 232165_at | 83481 | **EPPK1** |  |  | -0,60 |  |
| 202796_at | 11346 | **SYNPO** |  |  | -0,61 |  |
| 203216_s_at | 4646 | **MYO6** | -0,53 | -0,55 | -0,56 |  |
| NuGO_eht0306149_at | 27254 | **CSDC2** |  | -0,53 |  |  |
| 239024_at | 7707 | **ZNF148** |  |  | -0,73 |  |
| NuGO_eht0266505_at | 89869 | **PLCZ1** |  | 0,54 |  |  |
| 205034_at | 9134 | **CCNE2** |  | -0,50 |  |  |
| 209519_at | 4686 | **NCBP1** |  | -0,73 |  |  |
| 214433_s_at | 8991 | **SELENBP1** |  |  | -0,52 |  |
| 205134_s_at | 26747 | **NUFIP1** |  | 0,58 |  |  |
| 218158_s_at | 26060 | **APPL1** |  |  | -0,54 |  |
| 204420_at | 8061 | **FOSL1** |  |  | 0,72 |  |
| 203881_s_at | 1756 | **DMD** |  |  | 0,60 |  |
| 226181_at | 51175 | **TUBE1** |  |  | 1,04 |  |
| 219961_s_at | 55857 | **PLK1S1** |  |  | -0,54 |  |
| 209118_s_at | 7846 | **TUBA1A** |  | -0,53 |  |  |
| 212372_at | 4628 | **MYH10** |  |  | -0,56 |  |
| 212458_at | 200734 | **SPRED2** |  | 0,64 | 0,68 |  |
| 212675_s_at | 23177 | **CEP68** |  |  | -0,50 |  |
| 204011_at | 10253 | **SPRY2** |  |  | 0,56 |  |
| 204487_s_at | 3784 | **KCNQ1** |  | -0,69 | -0,58 |  |
| 200862_at | 1718 | **DHCR24** | -0,74 | -1,35 | -1,39 |  |
| 201791_s_at | 1717 | **DHCR7** | -0,53 |  | -0,71 |  |
| 202375_at | 9871 | **SEC24D** |  |  | 0,73 |  |
| 208647_at | 2222 | **FDFT1** | -0,55 | -0,79 | -0,89 |  |
| 202540_s_at | 3156 | **HMGCR** |  | -0,81 | -0,81 |  |
| 209218_at | 6713 | **SQLE** |  | -0,51 | -0,65 |  |
| 201626_at | 3638 | **INSIG1** | -1,87 | -2,86 | -2,99 |  |
| 202218_s_at | 9415 | **FADS2** | -0,58 | -0,71 | -0,74 |  |
| 223325_at | 51061 | **TXNDC11** |  |  | -0,53 |  |
| 214109_at | 987 | **LRBA** |  |  | -0,50 |  |
| 227759_at | 255738 | **PCSK9** |  | -0,53 | -0,56 |  |
| 203476_at | 7162 | **TPBG** |  | 0,69 | 0,60 |  |
| 226704_at | 118424 | **UBE2J2** |  |  | 0,55 |  |
| 225847_at | 57552 | **NCEH1** |  |  | 0,55 |  |
| 229549_at | 813 | **CALU** |  | 0,70 |  |  |
| NuGO_eht0331597_at | 283208 | **P4HA3** |  | 0,54 |  |  |
| 218436_at | 64374 | **SIL1** |  | 0,59 |  |  |
| 201650_at | 3880 | **KRT19** |  | -0,58 | -0,74 |  |
| 221276_s_at | 81493 | **SYNC** |  | -0,55 |  |  |
| 202068_s_at | 3949 | **LDLR** | -1,30 | -2,20 | -2,29 |  |
| 208881_x_at | 3422 | **IDI1** | -0,85 | -1,12 | -1,43 |  |
| 225826_at | 326625 | **MMAB** | -0,55 | -0,76 | -0,55 |  |
| 238427_at | 134266 | **GRPEL2** | 0,92 |  | 0,67 |  |
| 219174_at | 80173 | **IFT74** |  | -0,52 |  |  |
| 209018_s_at | 65018 | **PINK1** |  | -0,53 |  |  |
| 1552546_a_at | 137994 | **LETM2** |  | 0,62 | 0,94 |  |
| 203397_s_at | 2591 | **GALNT3** |  | 0,56 | 0,64 |  |
| 209946_at | 7424 | **VEGFC** |  | 0,86 | 1,05 |  |
| 213849_s_at | 5521 | **PPP2R2B** |  | -0,80 | -0,76 |  |
| 212218_s_at | 2194 | **FASN** |  | -0,52 |  |  |
| 202022_at | 230 | **ALDOC** |  |  | -0,62 |  |
| 1555037_a_at | 3417 | **IDH1** |  |  | -0,56 |  |
| 224391_s_at | 54414 | **SIAE** |  | -0,69 |  |  |
| 202847_at | 5106 | **PCK2** |  | 1,11 | 0,56 |  |
| 203989_x_at | 2149 | **F2R** |  | -0,64 |  |  |
| 203188_at | 11041 | **B3GNT1** |  |  | -0,56 |  |
| 55093_at | 54480 | **CHPF2** |  | 0,70 |  |  |
| 1554741_s_at | 387628 | **KGFLP1** |  | -0,54 |  |  |
| 204873_at | 5189 | **PEX1** |  | -0,53 |  |  |
| 204364_s_at | 65055 | **REEP1** |  |  | -0,70 |  |
| 208998_at | 7351 | **UCP2** |  |  | -0,60 |  |
| 206141_at | 27304 | **MOCS3** |  |  | 0,68 |  |
| 224436_s_at | 25934 | **NIPSNAP3A** |  | -0,55 |  |  |
| 214909_s_at | 23564 | **DDAH2** |  | -0,69 | -0,61 |  |
| 228499_at | 5210 | **PFKFB4** |  |  | -0,51 |  |
| 203708_at | 5142 | **PDE4B** |  |  | -0,52 |  |
| 212775_at | 23363 | **OBSL1** |  |  | -0,73 |  |
| 205191_at | 6102 | **RP2** | -0,61 | -0,55 |  |  |
| 209146_at | 6307 | **MSMO1** | -0,58 | -0,77 | -0,80 |  |
| 204568_at | 22863 | **ATG14** | -0,56 |  |  |  |
| 234488_s_at | 64396 | **GMCL1P1** | -0,55 |  |  |  |
| 1554242_a_at | 1690 | **COCH** | -0,50 |  |  |  |
| 237513_at | 136541 | **PRSS58** | 0,53 |  |  |  |
| 218303_x_at | 51315 | **KRCC1** |  |  | -0,62 |  |
| 242093_at | 94122 | **SYTL5** |  |  | -0,60 |  |
| 213075_at | 169611 | **OLFML2A** |  | -0,63 | -1,00 |  |
| 202388_at | 5997 | **RGS2** |  |  | 0,59 |  |
| 209270_at | 3914 | **LAMB3** |  | -0,53 |  |  |
| NuGO_eht0343916_s_at | 1299 | **COL9A3** |  | -0,63 | -0,63 |  |
| 1554547_at | 220965 | **FAM13C** |  | -0,90 | -0,61 |  |
| 205226_at | 5157 | **PDGFRL** |  | 0,66 | 0,52 |  |
| NuGO_eht0294668_at | 127002 | **ATXN7L2** |  |  | 0,52 |  |
| 243093_at | 79074 | **C2orf49** |  |  | 0,60 |  |
| 1552927_at | 257397 | **TAB3** |  | -0,59 |  |  |
| 206698_at | 7504 | **XK** |  | 0,84 | 0,53 |  |
| 217783_s_at | 51646 | **YPEL5** |  |  | -0,51 |  |
| NuGO_eht0344027_at | 344148 | **NCKAP5** |  | -0,71 |  |  |
| 220009_at | 79836 | **LONRF3** |  |  | 0,53 |  |
| 224443_at | 84791 | **LINC00467** |  | 0,70 |  |  |
| 224519_at | 100132167 | **LOC100132167** |  |  | 0,55 |  |
| 202667_s_at | 7922 | **SLC39A7** |  | 0,57 | 0,62 |  |
| 223315_at | 59277 | **NTN4** |  | 0,87 | 1,14 |  |
| 230670_at | 285313 | **IGSF10** |  |  | -0,58 |  |
| 238533_at | 2045 | **EPHA7** |  | -0,56 |  |  |
| NuGO_eht0282884_at | 11228 | **RASSF8** |  |  | 0,63 |  |
| 1559265_at | 387640 | **C10orf140** |  |  | -0,52 |  |
| 206547_s_at | 5475 | **PPEF1** |  |  | 0,54 |  |
| 212062_at | 10079 | **ATP9A** |  |  | -0,58 |  |
| 221599_at | 28971 | **C11orf67** |  |  | -0,66 |  |
| 201397_at | 26227 | **PHGDH** |  | 0,52 |  |  |
| 223533_at | 84230 | **LRRC8C** |  | 0,55 |  |  |
| 230748_at | 9120 | **SLC16A6** |  | 0,89 | 0,85 |  |
| 218451_at | 64866 | **CDCP1** |  | 0,65 |  |  |
| NuGO_eht0339798_at | 285696 | **LOC285696** |  |  | -0,52 |  |
| 220173_at | 80127 | **C14orf45** |  |  | 0,55 |  |
| 227526_at | 50937 | **CDON** |  | -0,53 |  |  |
| 214691_x_at | 54629 | **FAM63B** |  |  | 0,54 |  |
| 1552694_at | 114134 | **SLC2A13** |  | -0,57 |  |  |
| 224852_at | 55761 | **TTC17** |  |  | 0,76 |  |
| 226370_at | 80311 | **KLHL15** |  |  | -0,56 |  |
| 222549_at | 9076 | **CLDN1** |  | 0,87 | 0,84 |  |
| 212646_at | 23180 | **RFTN1** |  | 0,54 |  |  |
| 1553955_at | 129285 | **PPP1R21** |  |  | -0,61 |  |
| 218723_s_at | 28984 | **RGCC** |  |  | -0,83 |  |
| 225355_at | 54492 | **NEURL1B** |  |  | -0,60 |  |
| 203438_at | 8614 | **STC2** |  | 0,84 | 0,96 |  |
| 212830_at | 1955 | **MEGF9** |  |  | -0,56 |  |
| NuGO_eht0264563_at | 3589 | **IL11** |  | 0,73 | 1,13 |  |
| 213664_at | 6505 | **SLC1A1** |  | 0,67 | 0,67 |  |
| 213212_x_at | 374650 | **GOLGA6L5** |  |  | 0,58 |  |
| 220892_s_at | 29968 | **PSAT1** |  | 0,50 |  |  |
| 228186_s_at | 84870 | **RSPO3** |  | 0,56 |  |  |
| 206805_at | 10371 | **SEMA3A** |  |  | 0,57 |  |
| 219014_at | 51316 | **PLAC8** |  | -0,55 |  |  |
| 202562_s_at | 11161 | **C14orf1** |  | -0,54 |  |  |
| 222835_at | 79875 | **THSD4** |  | 0,55 |  |  |
| 211596_s_at | 26018 | **LRIG1** |  |  | 0,74 |  |
| NuGO_eht0294656_at | 284611 | **FAM102B** |  | -1,02 |  |  |
| 225911_at | 255743 | **NPNT** |  | -0,50 |  |  |
| 238931_at | 79066 | **METTL16** |  |  | 0,63 |  |
| 218674_at | 80006 | **C5orf44** |  | -0,75 |  |  |
| 225134_at | 84926 | **SPRYD3** |  | 0,63 |  |  |
| 218469_at | 26585 | **GREM1** |  |  | 0,60 |  |
| NuGO_eht0333542_s_at | 153830 | **RNF145** |  | -0,56 |  |  |
| 224321_at | 23671 | **TMEFF2** |  | 0,51 |  |  |
| 226150_at | 84513 | **PPAPDC1B** |  | 0,96 | 0,71 |  |
| 242871_at | 54852 | **PAQR5** |  |  | 0,54 |  |
| 227037_at | 201164 | **PLD6** |  | 0,74 | 0,59 |  |
| 228073_at | 140838 | **NANP** |  | 0,53 |  |  |
| 210041_s_at | 5238 | **PGM3** |  |  | 0,60 |  |
| 208978_at | 1397 | **CRIP2** |  | -0,60 |  |  |
| 205206_at | 3730 | **KAL1** |  |  | -0,64 |  |
| 225582_at | 85450 | **ITPRIP** |  | 0,68 | 0,58 |  |
| 202481_at | 9249 | **DHRS3** |  | 0,96 |  |  |
| 220770_s_at | 63920 | **C5orf54** |  |  | -0,52 |  |
| 221208_s_at | 79684 | **MSANTD2** |  | -0,71 | -0,55 |  |
| 226121_at | 147015 | **DHRS13** |  | 0,71 |  |  |
| 219296_at | 54503 | **ZDHHC13** |  | 0,51 |  |  |
| 224325_at | 8325 | **FZD8** |  | 0,63 |  |  |
| 226302_at | 5205 | **ATP8B1** |  | 0,58 |  |  |
| 219973_at | 79642 | **ARSJ** |  |  | 0,52 |  |
| 212136_at | 493 | **ATP2B4** |  | -0,57 |  |  |
| 213424_at | 23366 | **KIAA0895** |  | -0,89 |  |  |
| 218706_s_at | 65983 | **GRAMD3** |  | -0,62 | -0,68 |  |
| 243995_at | 375743 | **PTAR1** |  | -0,63 |  |  |
| 1554057_at | 645676 | **LOC645676** |  |  | -0,54 |  |
| 207761_s_at | 25840 | **METTL7A** |  | -0,86 | -1,08 |  |
| 1553960_at | 90203 | **SNX21** |  |  | -0,55 |  |
| 212419_at | 219654 | **ZCCHC24** |  |  | -0,65 |  |
| 226757_at | 3433 | **IFIT2** |  |  | 0,68 |  |
