# Supplementary figures and images for: Tocotrienols induce endoplasmic reticulum stress and apoptosis in cervical cancer cells
Source: Genes Nutr. 2016 Dec 23;11:32. doi: 10.1186/s12263-016-0543-1 (PMC5180413; doi:10.1186/s12263-016-0543-1)

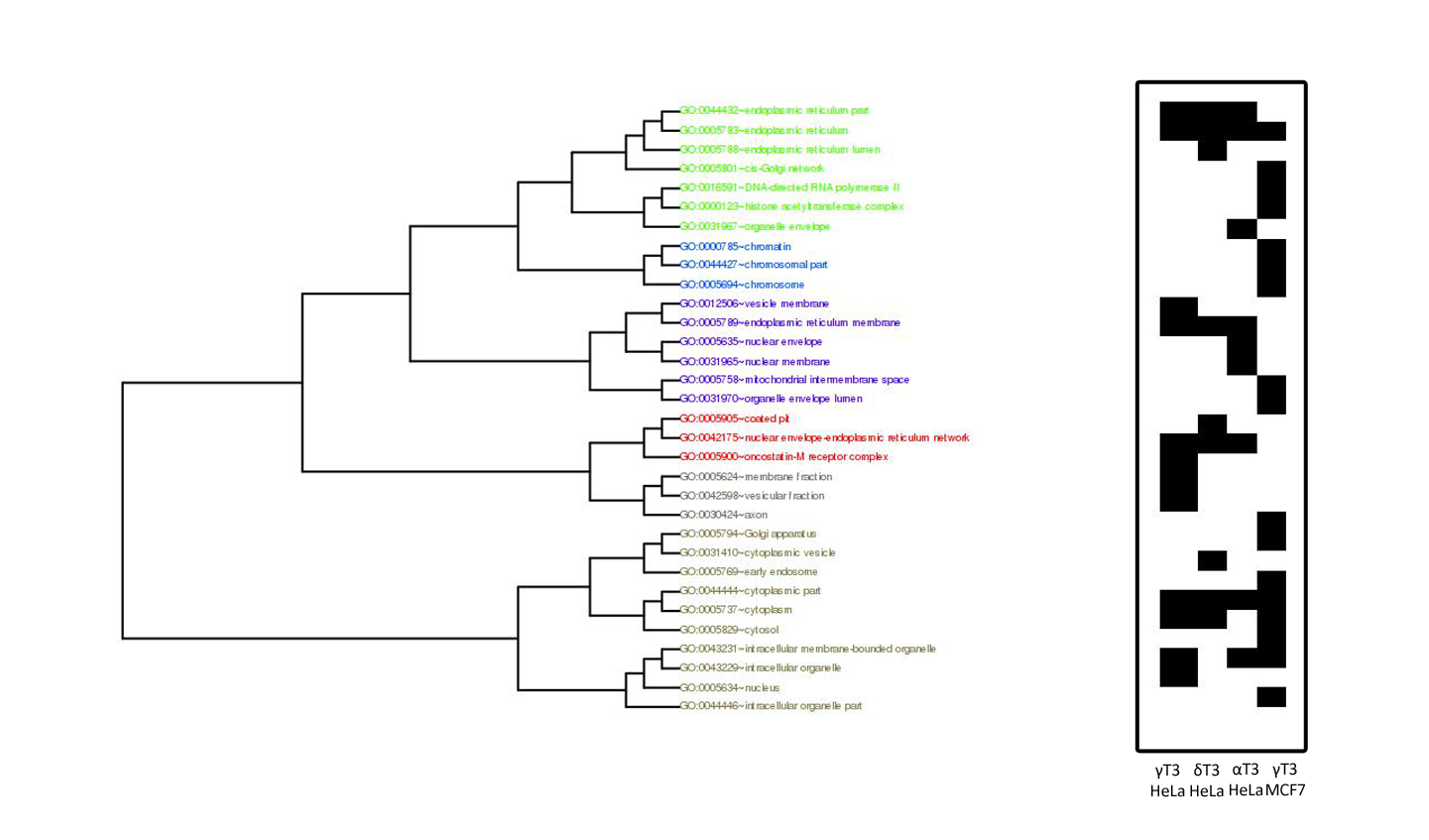

Supplement: Additional file 2: Figure S1. — Clusterization of CC enriched after T3 treatment in HeLa and MCF-7 cells. The distances between enriched CC were estimated according to the Resnik measure. The optimal number of clusters was estimated by silhouette scores and represented with different colors within the dendrogram. The box on the right side of the figure shows CCs enriched by a specific treatment. (TIF 3584 kb) [file 12263_2016_543_MOESM2_ESM.tif]

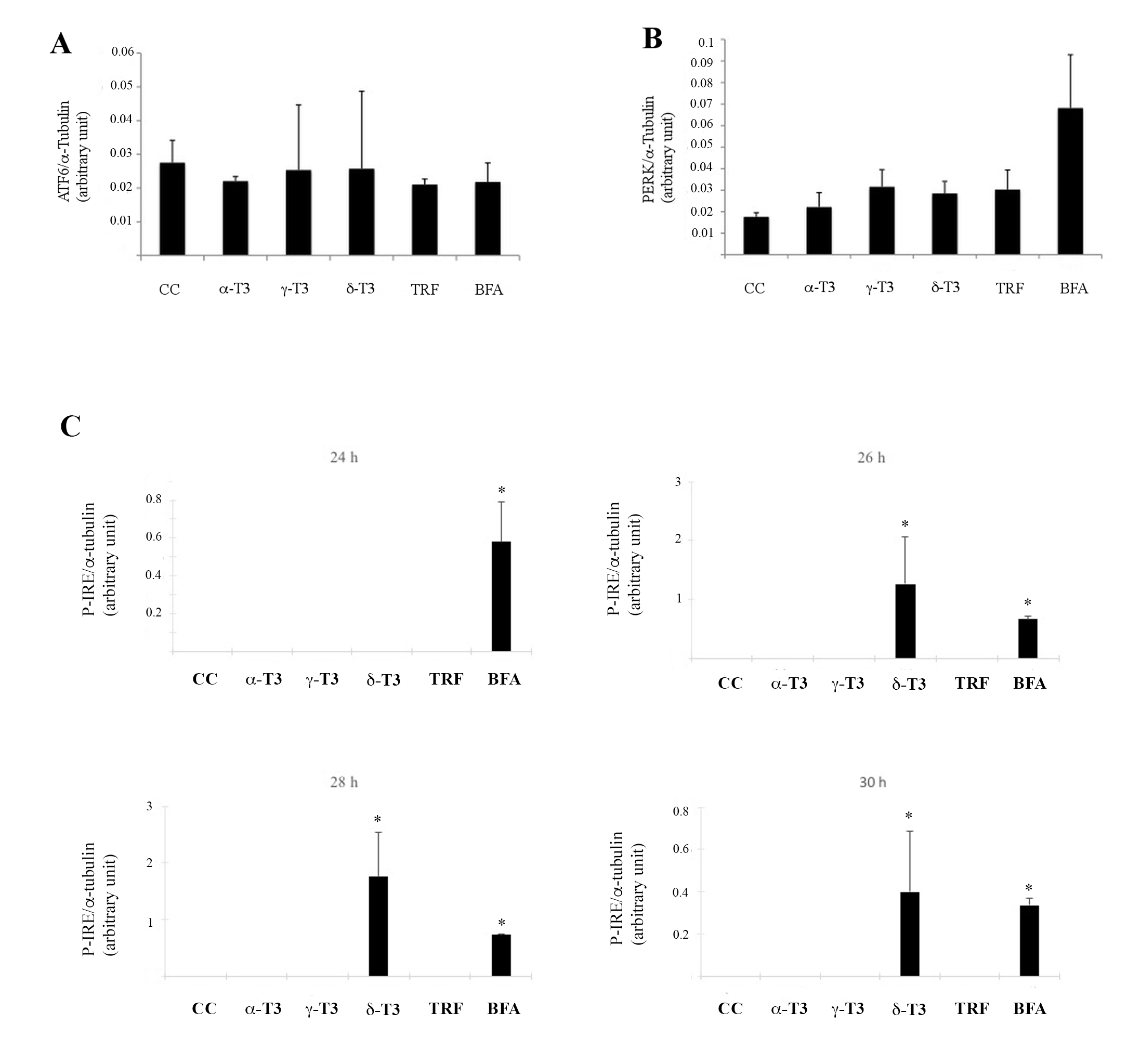

Supplement: Additional file 3: Figure S2. — Densitometric analysis of ATF-6, PERK, and IRE-1α activity. Treatments with T3 have no significant effect on the protein expression of ATF-6 and PERK at 24 h from the treatment. Conversely, IRE-1 phosphorylation is significantly affected by T3 treatment. Data were analyzed by one-way ANOVA with repeated measures followed by Fisher’s test. Asterisks indicate significant differences (p value ≤0.05) between treated cells vs control (CC). (TIF 13186 kb) [file 12263_2016_543_MOESM3_ESM.tif]
